# Integrated optimization of unmanned aerial vehicle task allocation and path planning under steady wind

**DOI:** 10.1371/journal.pone.0194690

**Published:** 2018-03-21

**Authors:** He Luo, Zhengzheng Liang, Moning Zhu, Xiaoxuan Hu, Guoqiang Wang

**Affiliations:** 1 School of Management, Hefei, Anhui, China; 2 Key Laboratory of Process Optimization & Intelligent Decision-making, Ministry of Education, Hefei, Anhui, China; Nanyang Technological University, SINGAPORE

## Abstract

Wind has a significant effect on the control of fixed-wing unmanned aerial vehicles (UAVs), resulting in changes in their ground speed and direction, which has an important influence on the results of integrated optimization of UAV task allocation and path planning. The objective of this integrated optimization problem changes from minimizing flight distance to minimizing flight time. In this study, the Euclidean distance between any two targets is expanded to the Dubins path length, considering the minimum turning radius of fixed-wing UAVs. According to the vector relationship between wind speed, UAV airspeed, and UAV ground speed, a method is proposed to calculate the flight time of UAV between targets. On this basis, a variable-speed Dubins path vehicle routing problem (VS-DP-VRP) model is established with the purpose of minimizing the time required for UAVs to visit all the targets and return to the starting point. By designing a crossover operator and mutation operator, the genetic algorithm is used to solve the model, the results of which show that an effective UAV task allocation and path planning solution under steady wind can be provided.

## Introduction

At present, unmanned aerial vehicles (UAVs) are widely used by the military and civilians. They are able to fulfill a variety of tasks, such as target reconnaissance [1], target tracking [2], intelligence gathering [3], post-earthquake rescue [4], and geological exploration [5]. For instance, when multiple UAVs are applied to cooperative target reconnaissance, it is necessary to allocate reconnaissance targets for each UAV reasonably, and to plan optimal flight path for each UAV. This involves the integrated optimization of task allocation and path planning constrained by multiple factors [6–8], which is also a NP-hard (non-deterministic polynomial-time hard) problem [9].

For UAVs, one of the largest disturbances in flight is wind, as it affects the flight posture [10] and flight path [11] of UAV, thus resulting in changes in flight time. In a windless environment, UAV airspeed is equivalent to UAV ground speed. However, in a windy environment, ground speed is affected by both airspeed and wind speed. Therefore, although UAV can fly along the pre-planned path in a windy environment by adjusting its flight speed and heading angle, the actual arrival time and fuel consumption will be higher than estimated [12], and the flight time will no longer be the shortest.

The integrated optimization of UAV task allocation and path planning under steady wind can be described as follows: In a windy environment, UAVs depart from the common starting point to visit multiple targets, and each target can only be visited once by one UAV. Constrained by factors such as physical characteristics [13] of UAV, the task allocation scheme and the optimal flight path are obtained, which enable UAVs to complete all the tasks and return to the starting point in the shortest time. In this paper, based on the classical vehicle routing problem (VRP) model, a variable-speed Dubins path vehicle routing problem (VS-DP-VRP) model is established with minimum flight time as the objective function. The following two factors are taken into account in the modeling:

First, given the flight dynamics constraints of UAV, the VRP model is extended to a Dubins path VRP model (DP-VRP). When flying between different targets, UAVs are constrained by flight dynamics and must change flight direction with the minimum turning radius. Thus, the obtained flight distance is not the Euclidean distance, but rather the Dubins distance. In addition, the Euclidean distance between two targets is constant in the VRP model, but the Dubins distance between the two targets in the DP-VRP model will change with the angle when a UAV visits the target.

Second, in light of the influences caused by wind, the DP-VRP model is further extended to the VS-DP-VRP model. In the standard VRP model, the time of a vehicle moving between two targets is constant or quantitatively changes according to the traffic conditions between the two points [14]. In the VS-DP-VRP model, on the other hand, because of the real-time changes in direction of UAV airspeed vector, as well as in the ground speed vector affected by both airspeed and wind speed, flight time of UAV between two targets is determined by real-time ground speed and Dubins distance between the two targets.

Currently, the heuristic algorithm is used to solve the VRP model. In particular, the genetic algorithm (GA) has been proved to be effective [15] in solving the bench mark dataset, and better results can be obtained by adjusting parameters. When solving practical problems, the GA has also been used as an efficient algorithm with which to solve the problem of task allocation and path planning [9]. In most cases, it is better than other algorithms [16] and requires shorter computation time [17]. In this paper, based on the GA framework, the VS-DP-VRP model is solved by designing a crossover operator and mutation operator.

The remainder of this paper is organized as follows: research related to this problem is introduced in the next section, and the VS-DP-VRP model is presented in the Section 3. Then, a GA is proposed for solving the VS-DP-VRP in Section 4. The model and algorithm are analyzed using example comparison in Section 5 and the parameter sensitivity experiment and simulation experiments are described in Section 6. Conclusions and the future prospects of this study are provided in Section 7.

## Related work

When UAVs are on a mission, the impact exerted by wind cannot be ignored, especially in terms of UAV task allocation and path planning [18, 19], because wind changes flight time and flight path [20–22] of UAV. At present, the modeling of wind can be divided into three ways: the first is the steady wind, which is of constant speed and direction [23–25]; the second is to model the wind according to the cause of wind [26], such as pressure, temperature, humidity, terrain, altitude, etc.; the third way is to estimate the status data of the current wind by analyzing historical data. Among these three methods, the steady wind is the typical one chosen for UAVs. It is believed that the speed and direction of wind influencing UAVs are constant. Aiming at UAV path planning in the steady wind, Zhang et al. [25] took into account the impact of steady wind on flight path of UAV, and set a virtual target, then the problem became the path planning for UAV that would cost the least time to reach the virtual target in a windless environment. Techy et al. [27] suggested linking straight and trochoidal path segments to form a feasible path and choosing the scheme that costs the least time from the result set.

Actually, when UAVs are in a steady wind, if they have a constant airspeed, their actual speed and direction are always changing relative to the ground [27], as they are under the influence of wind. That means their ground speed is changing all the time. In this case actual flight path of UAV no longer meets the estimated result of the plan and flight time changes as well. To keep the constant ground speed of UAV, airspeed and direction must be changing constantly under steady wind according to the vector relation [28] between UAV ground speed and airspeed. Although it can be guaranteed that UAV will fly along the pre-planned route, flight time changes. Thus, in steady wind, the time UAVs require to accomplish the task does not conform to the pre-planned time, no matter which flight control strategy is adopted. Affected by the wind, the optimization objective of UAV task allocation and path planning is to minimize execution time.

At present, in terms of UAV task allocation and path planning, the travelling salesman problem (TSP) model, team orienteering problem (TOP) model, and VRP model are usually adopted for the modeling. In particular, when all targets need to be visited, the optimization is often to minimize flight path, and the TSP model can be used for modeling. For example, Sathyan et al. [29] changed the UAV task allocation problem to the multiple travelling salesman problem (MTSP), and put forward a cluster-first approach in which each UAV is distributed to a target subset. Ernest et al. [30] extended the MTSP of multiple UAVs visiting multiple sites to the multi-depot polygon visiting Dubins MTSP, with a view to accounting for the constraint of UAV minimum turning radius.

However, in the case in which not all targets need be visited, the TOP model can be used to transfer the problem of the shortest flight path to the problem of maximizing profits. For example, Evers et al. [31] established a standard orienteering problem (OP) extended model, with a view of time windows and time-sensitive targets, with the purpose of solving the UAVs task allocation problem. Considering the impact on UAV flight time caused by sensor allocation, Mufalli et al. [32] established a TOP model to maximize the profits. The VRP model is generally used for modeling in consideration of the time used by UAVs to visit all targets. In a study conducted by Faie et al. [33], a VRP model was established with minimum flight time of each UAV as the cost function. In the meantime, it was pointed out that in the cases where wind could be ignored, the minimum flight time of UAV has the same meaning as the minimum path length. Guerrero J A et al. [19] took into account the wind and UAV energy constraints, and established a capacitated vehicle routing problem (CVRP) model targeting time minimization.

In the VRP standard model and extended model [34], it is believed that targets and their distances are constant. However, constrained by flight dynamics like minimum turning radius, the shortest flight distance of UAV is the length of the Dubins path rather than the Euclidean distance. The Dubins path is the feasible path [35] of minimum length, which moves along a bounded curve path with a constant speed. Moreover, it is a UAV’s shortest flight path in either a windless [36, 37] or windy environment [38]. According to the method of calculating the Dubins path [36], if the ground speed heading angles when a UAV visits two targets are equally divided into 360 parts, there are 6×360×360 = 777600≈7×105 solutions in the Dubins path between these two targets. Therefore, optimizing the Dubins path on the basis of optimizing the standard VRP is required.

At present, integer programming or a heuristic method is generally adopted to solve the standard or extended VRP model, but the calculation time of integer programming grows exponentially with the expansion of scale. The solution can be quickly acquired on the premise of ensuring the quality of the solution by designing a heuristic algorithm [39]. The common heuristic algorithm includes the GA [40], tabu search (TS) [41], particle swarm optimization (PSO) [42–43], differential evolution (DE) [44] etc. Among these, the GA can obtain a good solution in a shorter time [45–47], especially in solving UAV task allocation [48] and path planning [49, 50]. The GA has obtained a better effect not only in experiments, but also in engineering applications [51, 52].

## Problem description and modeling

In this section, wind, UAVs, targets, airspeed, and ground speed are described, and an integrated optimization model of UAV task allocation and path planning under steady wind is established. The symbols and nomenclature are shown in Table 1.

**Table 1:** The nomenclature.

Nomenclature			
*$\vec{V_{w}}$*	wind vector	*r*_min_	minimum turning radius
*β*_*w*_	wind direction	*V*_*w*_	wind speed
*N*_*U*_	number of UAVs	*U*	cooperating fixed wing UAVs set
$\vec{x}$	a UAV’s X-axis in a Cartesian inertial reference system	$\vec{y}$	a UAV’s Y-axis in a Cartesian inertial reference system
$\vec{\Omega_{\max}}$	maximum angular velocity	*c*	steering command
$\vec{\beta_{g}}$	the angular velocity of ground speed	$\vec{\beta_{a}}$	the angular velocity of air speed
*β*_*g*_	heading angle of ground speed	*$\vec{V_{g}}$*	ground speed vector
*β*_*a*_	heading angle of air speed	$\vec{V_{a}^{}}$	air speed vector
*V*_*g*_	ground speed	*V*_*a*_	air speed
*N*_*r*_	number of targets	*T*	set of targets
*L*	turning to the left	*T*_0_	the common starting point
*S*	straight line motion	*R*	turning to the right
*H*	ground speed heading angle discretization set	*N*_*g*_	ground speed heading angle discretization coefficient
Δ*θ*	the angular rotation	*V*_*g*_(*θ*)	ground speed when UAV’s *β*_*g*_ = *θ*
$X_{\left(T_{j},\beta_{gj},T_{k},\beta_{gk}\right)}^{i}$	decision variable	$t_{\left(T_{j},\beta_{gj},T_{k},\beta_{gk}\right)}^{i}$	flight time
*J*	the objective function of VS-DP-VRP model		

### Wind

Let

$$\vec{V_{w}}=\left(V_{w},\beta_{w}\right)$$ (1)

be the wind vector. *V*_*w*_ denotes wind speed; that is, the distance wind travels relative to the ground in unit time. *β*_*w*_ denotes the direction of wind, which refers to the direction of ambient air’s motion, and it is described by angle.

**Fig 1:**
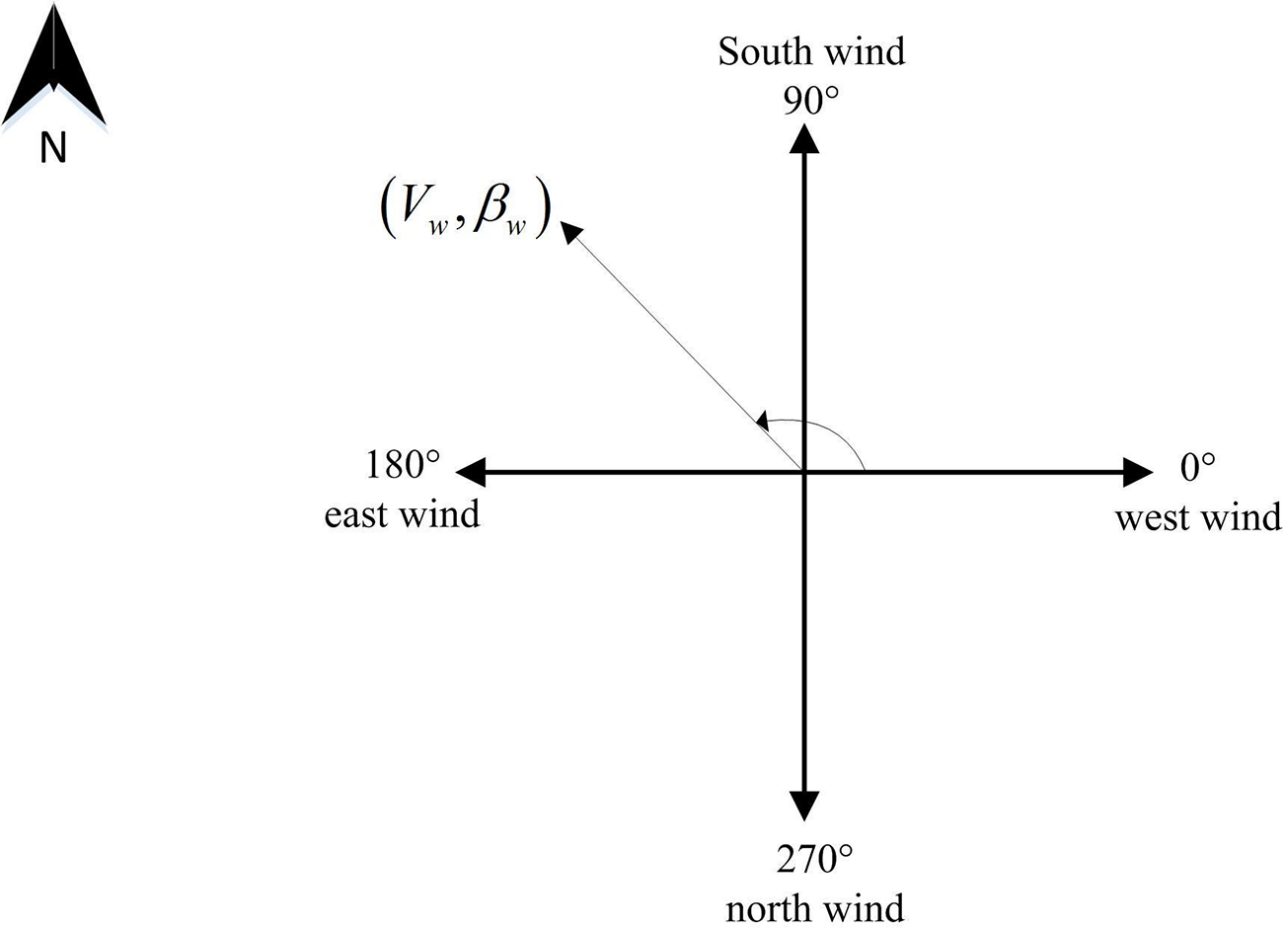
Wind direction.

In this paper, west wind (W) is defined as 0°, and south wind (S), east wind (E), and north wind (N), respectively, correspond to angles of 90°, 180°, and 270°, as depicted in Fig 1. Meanwhile, only the steady wind is taken into account. To be specific, the speed and direction of wind are constant in the given environment.

### UAVs

Let

$$U=\{U_{1},U_{2},\cdots ,U_{N_{U}}\}$$ (2)

be the set of *N*_*U*_ cooperating fixed wing UAVs. We assume that the vehicles spatial configuration can be defined by three states

$$q=\left(x,y,\beta_{a}\right)$$ (3)

with the following equations of motion:

$$\vec{x}=\vec{V_{g}}\cos\vec{\beta_{g}}$$ (4)

$$\vec{y}=\vec{V_{g}}\sin\vec{\beta_{g}}$$ (5)

$$\vec{\beta_{a}}=c\vec{\Omega_{\max}}$$ (6)

where $\vec{x}$ and $\vec{y}$ are a UAV’s coordinates in a Cartesian inertial reference system; $\vec{V_{g}}$ represents the ground speed of UAV; $\vec{\beta_{g}}$ denotes the heading angle of ground speed; ${\vec{\beta }}_{a}$ denotes the angular velocity of airspeed; c is the steering command, such that |*c*|≤1; and Ω_max_ is the maximum angular velocity of UAV. The definition and relationship between ${\vec{\beta }}_{g}$ and ${\vec{\beta }}_{a}$ will be explained in the Airspeed and ground speed section. Moreover, the UAVs mentioned in this paper also meet the following conditions: (1) all UAVs have the same minimum turning radius *r*_min_; (2) all UAVs are equipped with a collision-avoidance function; and (3) all UAVs fly at constant altitude with constant airspeed value.

### Targets

Let

$$T=\{T_{0},T_{1},T_{2},\cdots ,T_{N_{T}}\}$$ (7)

be the set of *N*_*T*_ targets, with known positions, and each point can only be visited once. denotes *T*_0_ the common starting point. According to Shima [8], continuous heading angle of UAV when visiting a target can be equally divided into 36 parts, which can guarantee accuracy and make the solving process less complex. Therefore, the ground speed heading angle discretization set is defined as

$$H=\left\{\beta_{gi};\beta_{gi}=\frac{2\pi i}{N_{g}},i=0,1,\ldots ,N_{g}-1\right\},\;N_{g}=36.$$ (8)

**Fig 2:**
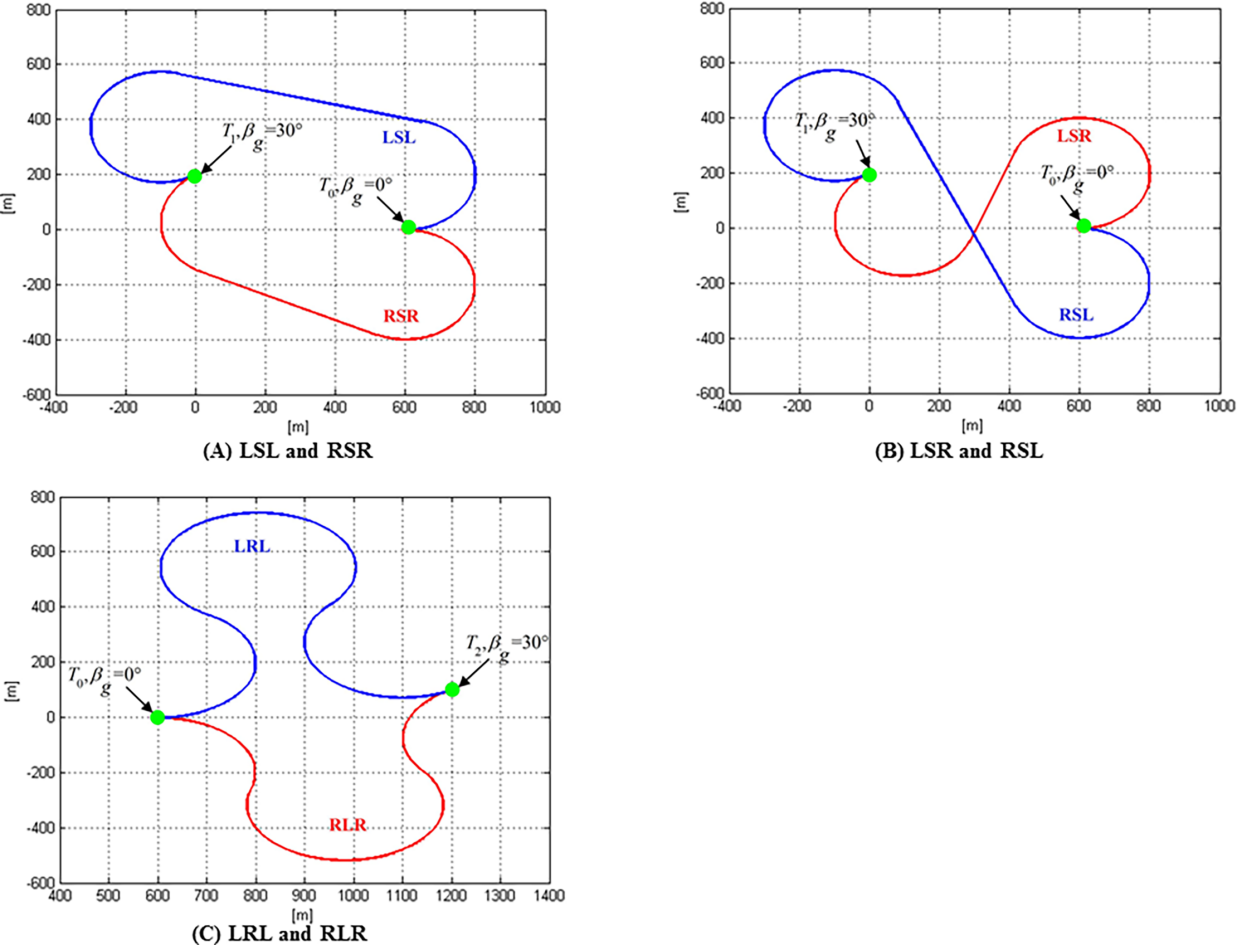
Dubins path.

The shortest distance between two targets is the Euclidean distance. However, constrained by flight dynamics, UAVs must be moved at the minimum turning radius, resulting in the actual flight path is Dubins path. According to the method of calculating the Dubins path, the Dubins path between two targets can be the combination of arc paths and straight path. There are six combinations [9], namely *D* = {*LSL*,*RSR*,*RSL*,*LSR*,*RLR*,*LRL*}. *L* represents an arc path of a UAV turning left with radius *r*_min_; *R* represents an arc path of a UAV turning right with radius *r*_min_; and *s* represents a UAV flying along a straight line. For example, when a UAV starts from *T*_0_ *with β*_*g*_ = 0° to *T*_1_ with *β*_*g*_ = 30°, all the possible Dubins paths can be described in Fig 2. With the changes in ground speed heading angles of two targets, there are a total of 6×36×36 = 7776 kinds of Dubins paths between the two targets.

### Airspeed and ground speed

Let

$$\vec{V_{a}}=(V_{a},\beta_{a}),$$ (9)

be the airspeed vector. *V*_*a*_ is the airspeed and *β*_*a*_ is the airspeed heading angle. In accordance with the flight envelope of UAV, there are upper and lower bounds [27] for its airspeed when flying at constant altitude with a constant load; namely, *V*_*a*_ ⊂[*V*_*a*_min_,*V*_*a*_max_]. *V*_*a*_min_ and *V*_*a*_max_ represent, respectively, the minimum and maximum value of the airspeed at the specified height.

Let

$$\vec{V_{g}}=(V_{g},\beta_{g}),$$ (10)

be the ground speed vector; that is, speed relative to the ground under the influence of wind. *V*_*g*_ is the ground speed and *β*_*g*_ denotes its heading angle. The ground speed vector can be obtained by formula (11) using the airspeed vector $\vec{V_{a}}$ and wind speed vector $\vec{V_{w}}$.

$$\left(\begin{array}{l} \cos\beta_{g}\\ \sin\beta_{g} \end{array}\right)V_{g}=\left(\begin{array}{c} \cos\beta_{a}\;\cos\beta_{w}\\ \sin\beta_{a}\;\sin\beta_{w} \end{array}\right)\left(\begin{array}{c} V_{a}\\ V_{w} \end{array}\right)$$ (11)

**Fig 3:**
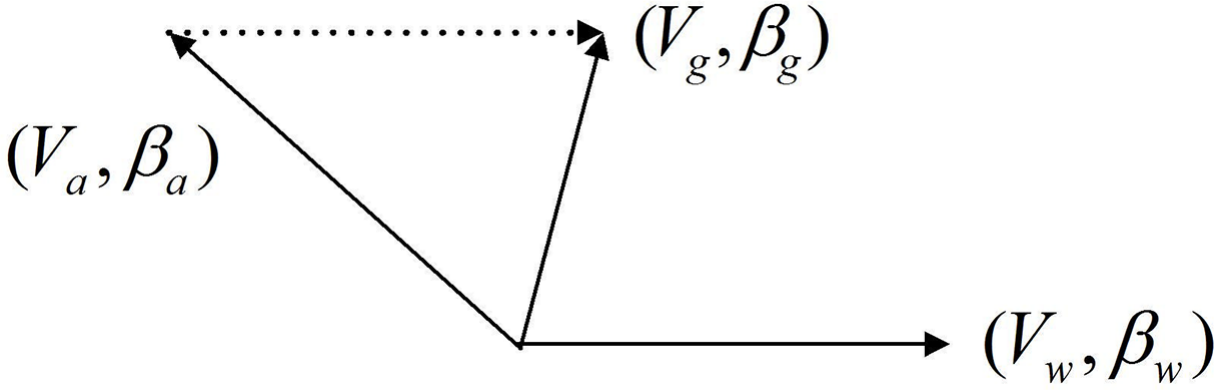
Relationship between speed vectors.

The relationship between the three speed vectors is shown in Fig 3. It can be easily seen that in a windless environment the airspeed of UAV is the same as the ground speed, namely *V*_*w*_ = 0*m*/*s*, $\vec{V_{a}^{}}=\vec{V_{g}^{}}$, and $\vec{\beta_{a}}=\vec{\beta_{g}}$.

**Fig 4:**
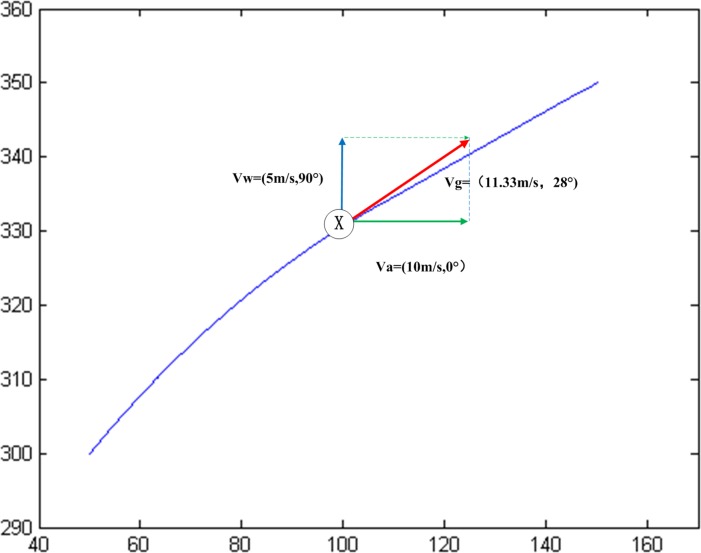
Ground speed vector at point X (100,330).

For instance, the ground speed vector of UAV at point X (100,330) when flying in a south wind can be obtained by formula (10), $\vec{V_{w}}=({11.33m/s,28}^{⊙})$, as shown in Fig 4.

The time used by UAV *U*_*i*_ departing from a target *T*_*j*_ at a ground speed heading angle *β*_*gj*_ to reach target *T*_*k*_ at a ground speed heading angle *β*_*gk*_ is calculated using

$$t_{\left(T_{j},\beta_{gj},T_{k},\beta_{gk}\right)}^{i}=\sum_{\theta =\beta_{gj}}^{\beta_{gk}}\int_{\theta }^{\theta +\Delta \theta }\frac{r_{}}{V_{g}\left(\theta \right)}d\theta ,\forall i\in \{1,\ldots {,N}_{U}\},\forall j,k\in \{0,\ldots ,N_{T}\}$$ (12)

where Δ*θ* is the angular rotation and *V*_*g*_(*θ*) is the ground speed when the UAV’s ground speed heading angle is *θ*.

A UAV’s actual flight time between two targets is determined by both the ground speed vector and the Dubins distance between the two targets.

### VS-DP-VRP modeling

The integrated optimization of UAV task allocation and path planning under steady wind is essentially the planning of order and angle for each UAV to visit a series of targets. In this paper, the VS-DP-VRP model is established with the minimum time used by UAVs to visit all targets and return to the starting point as the objective function:

$$J=\max\{\sum_{j=0}^{N_{T}}\sum_{k=0}^{N_{T}}X_{\left(T_{j},\beta_{gj},T_{k},\beta_{gk}\right)}^{i}t_{\left(T_{j},\beta_{gj},T_{k},\beta_{gk}\right)}^{i},i=1,\ldots ,N_{U}\},$$ (13)

$$X_{\left(T_{j},\beta_{gj},T_{k},\beta_{gk}\right)}^{i}\in \left\{0,1\right\},\forall i\in \left\{1,\cdots ,N_{U}\right\},\forall j,k\in \left\{0,\cdots ,N_{T}\right\},\forall gj,gk\in \{0,\cdots ,N_{g}-1\},$$ (14)

where $X_{\left(T_{j},\beta_{gj},T_{k},\beta_{gk}\right)}^{i}$ is the decision variable. If UAV *U*_*i*_ depart from target *T*_*j*_ at ground speed heading angle *β*_*gj*_ and reach target *T*_*k*_ at ground speed heading angle *β*_*gk*_, $X_{\left(T_{j},\beta_{gj},T_{k},\beta_{gk}\right)}^{i}=1$; otherwise, $X_{\left(T_{j},\beta_{gj},T_{k},\beta_{gk}\right)}^{i}=0$;

The constraints of the model are as follows:

1. Visit constraint for targets: All targets can be visited only once;
  $$\sum_{i=1}^{N_{U}}\sum_{k=1}^{N_{T}}\sum_{gk=0}^{N_{g}-1}\sum_{gj=0}^{N_{g}-1}X_{\left(T_{j},\beta_{gj},T_{k},\beta_{gk}\right)}^{i}=1,\forall j\in \{0,\ldots {,N}_{U}\}.$$ (15)
2. UAV path constraints: Each UAV departs from the starting point, and then returns to the starting point after visiting a number of targets;

$$\sum_{k=1}^{N_{T}}\sum_{gk=0}^{N_{g}-1}\sum_{i=1}^{N_{U}}X_{\left(T_{0},\beta_{g0},T_{k},\beta_{gk}\right)}^{i}=\sum_{k=1}^{N_{T}}\sum_{gk=0}^{N_{g}-1}\sum_{i=1}^{N_{U}}X_{\left(T_{k},\beta_{gk},T_{0},\beta_{g0}\right)}^{i}=N_{U},$$ (16)

$$\sum_{i=1}^{N_{U}}\sum_{j=0}^{N_{T}}\sum_{gj=0}^{N_{g}-1}\sum_{k=0}^{N_{T}}\sum_{gk=0}^{N_{g}-1}X_{\left(T_{j},\beta_{gj},T_{k},\beta_{gk}\right)}^{i}=N_{T}+N_{U}.$$ (17)

## GA-based optimization algorithm

By designing the crossover and mutation operator, the GA is used to solve the VS-DP-VRP model.

### Encoding

A chromosome represents a feasible solution to the problem. In this paper, a chromosome encodes by three parts: the targets, the ground speed heading angles and the UAVs, where targets belong to set *T*, ground speed heading angles belong to set *H* and UAVs belong to set *U*.

**Fig 5:**
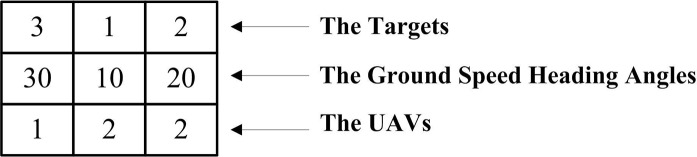
Encoding of chromosome.

The chromosome shown in Fig 5 represents a feasible solution for two UAVs to visit three targets. This feasible solution means *U1* departed from the starting point and then returned to the starting point after visiting *T3* with *β*_*g*_ = 300°, while *U2* departed from the starting point to visit *T1* with *β*_*g*_ = 100°, and then returned to the starting point after visiting *T2* with *β*_*g*_ = 200°.

### Crossover

**Fig 6:**
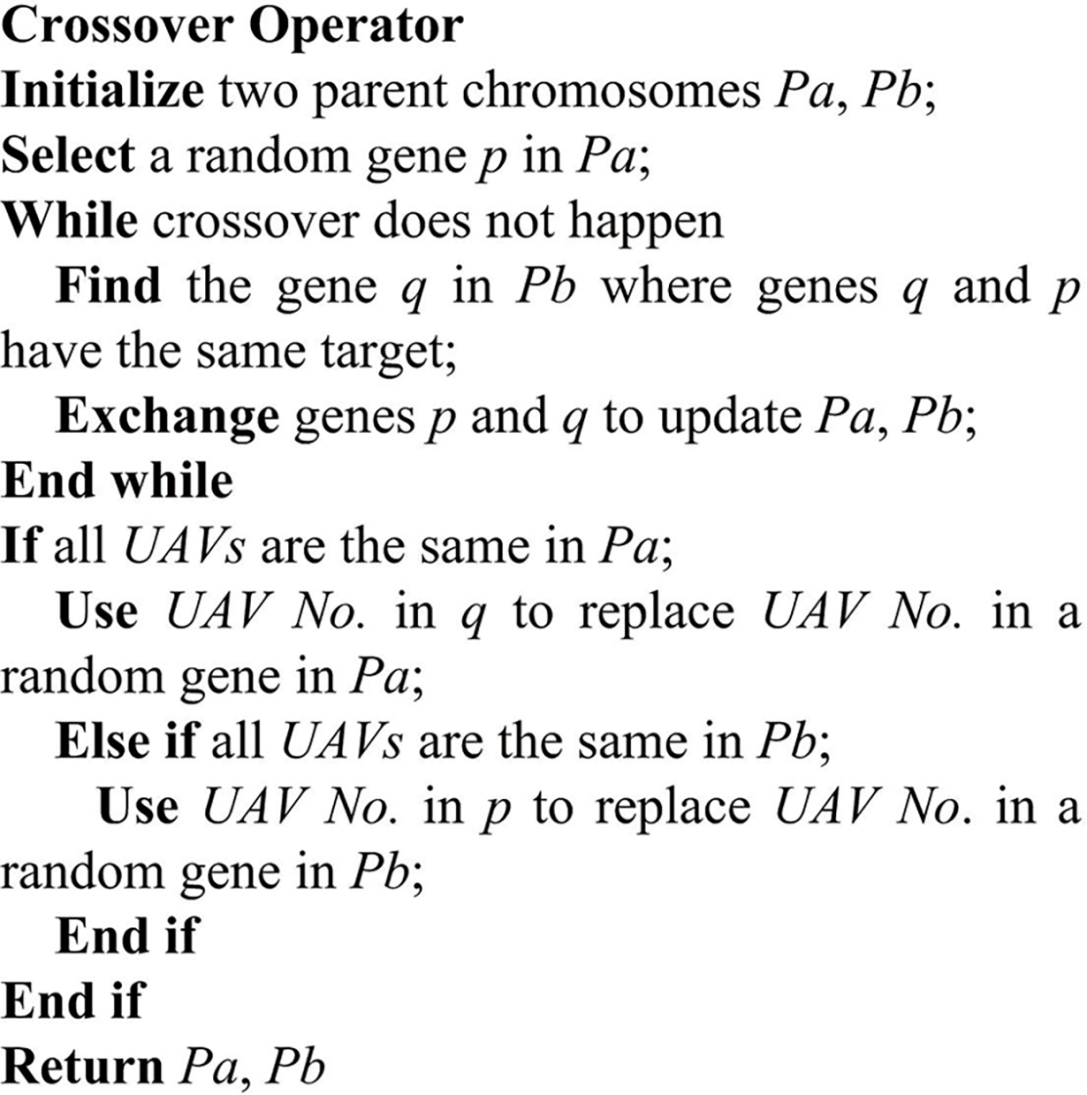
The pseudocode of crossover operator.

The population diversity can be increased by a crossover operation. For the VS-DP-VRP model, a new crossover operator is designed in this paper, and its pseudocode is shown in Fig 6.

**Fig 7:**
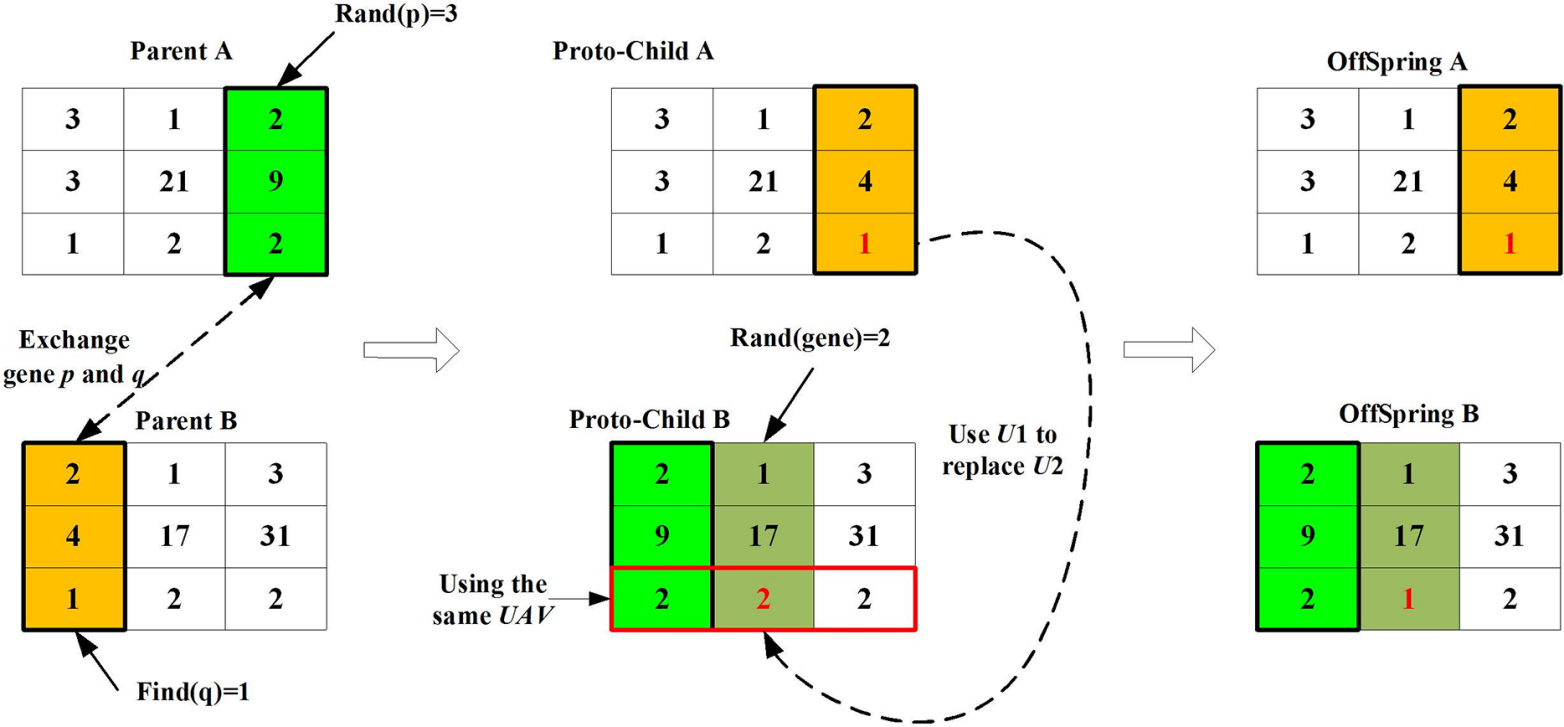
Crossover process of two chromosomes.

Fig 7 shows the crossover process of the parent chromosomes *Parent A* and *Parent B*. The third gene in *Parent A* is exchanged with the first gene in *Parent B* to generate two new chromosomes, namely *ProtoChild A* and *ProtoChild B*. Since all UAVs encoding in *ProtoChild B* are *U2*. In the next step, the second gene, which is selected randomly, in *ProtoChild B* is changed with the third gene in *ProtoChild A* to complete the cross operation to generate *OffSpring A* and *OffSpring B*.

### Mutation

**Fig 8:**
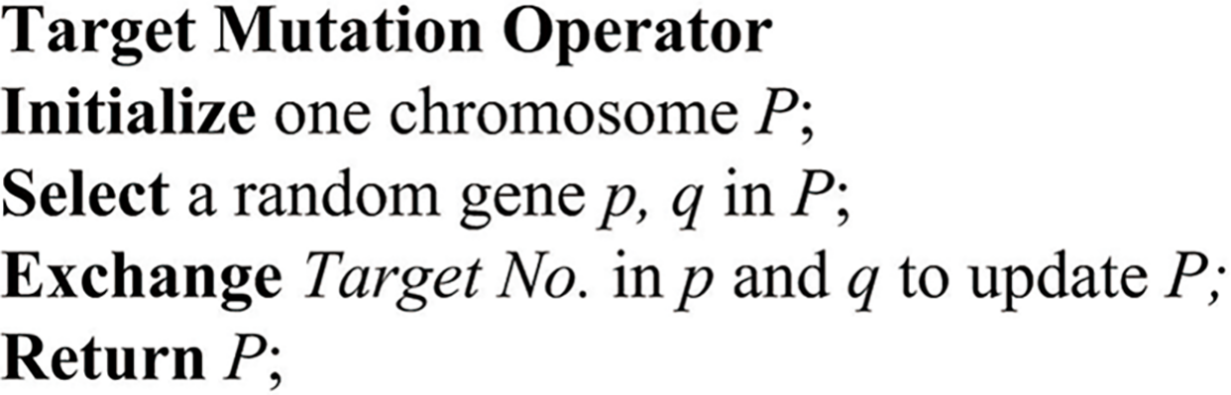
The pseudocode of target mutation operator.

**Fig 9:**
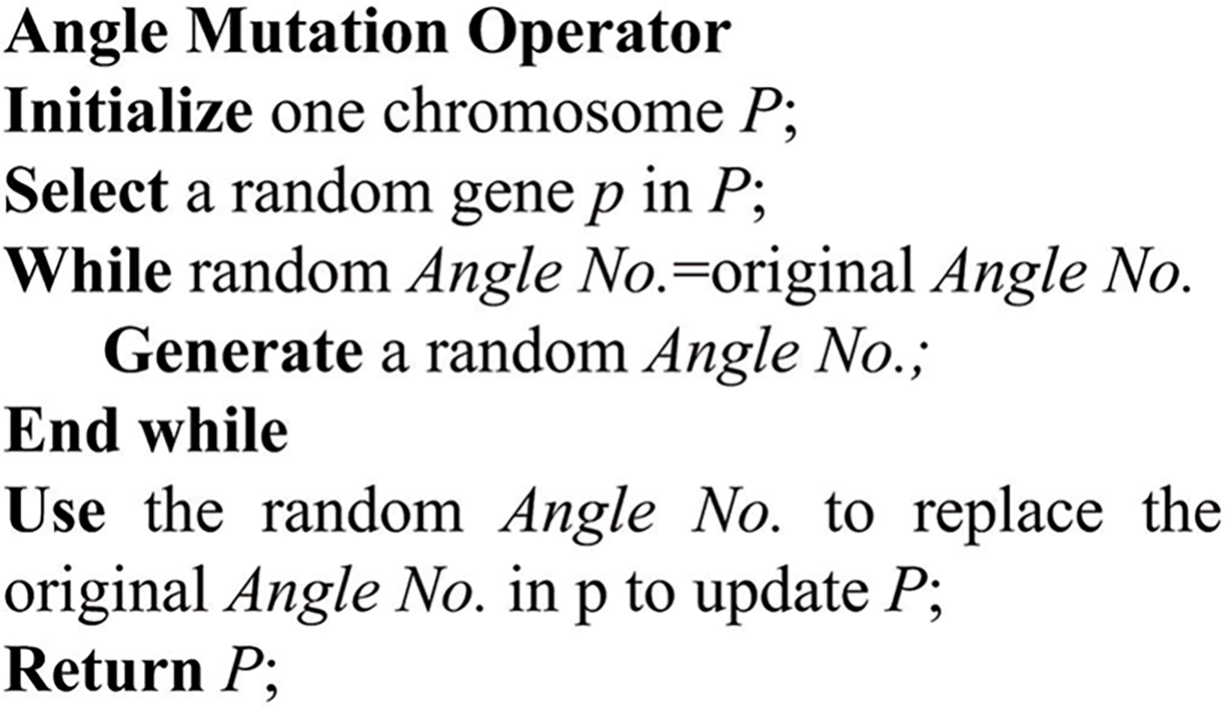
The pseudocode of angle mutation operator.

**Fig 10:**
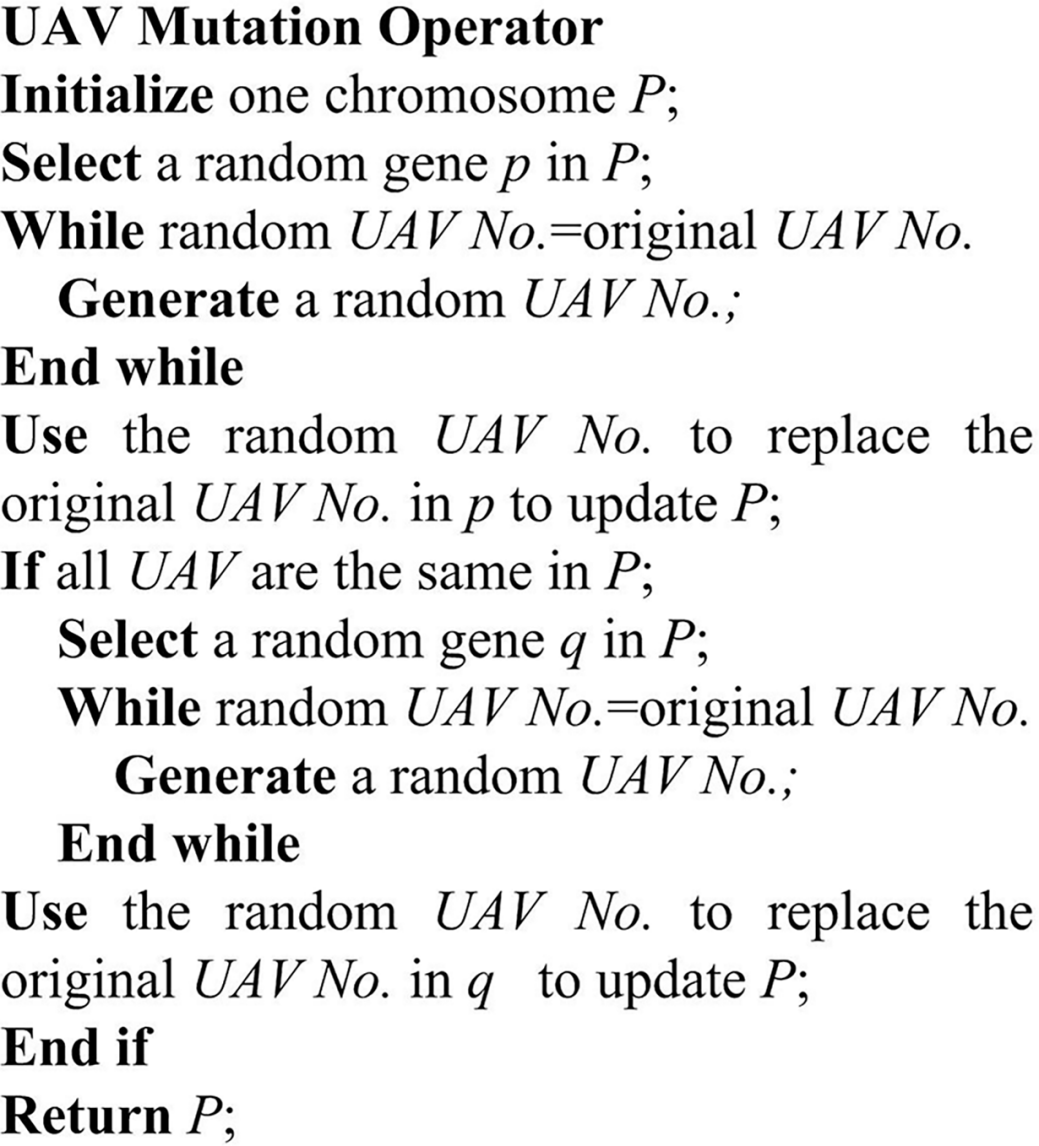
The pseudocode of UAV mutation operator.

Mutation can prevent the GA from local optimum. In this paper, three kinds of mutation operators are designed; namely, the mutation of targets, ground speed heading angle mutation, as well as mutation in UAV allocation. Since the above three mutations do not have a correlation, the roulette method has been used to determine whether the above mutations occurred. Moreover, we allow all three mutations to be applied on each chromosome concurrently. The pseudocode of the three kinds of mutation operators is shown in Figs 8–10.

**Fig 11:**
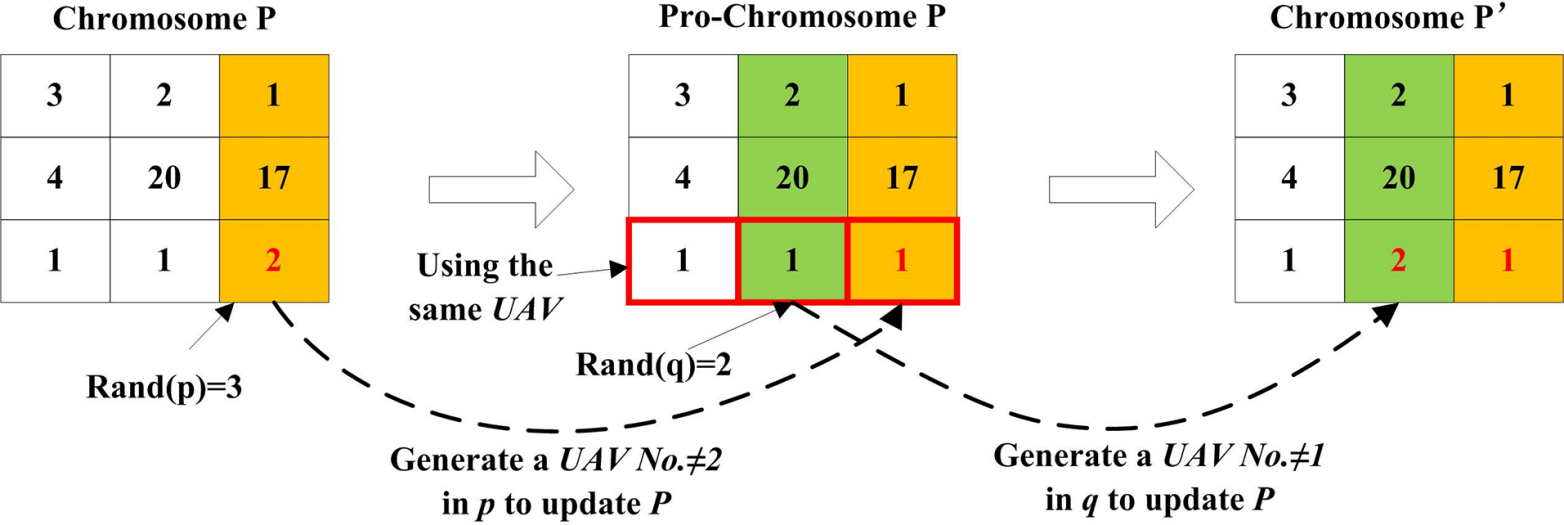
Process of UAV mutation for chromosome.

Fig 11 shows the procedure of selecting the third gene in chromosome *P* for the UAV mutation operator. Firstly, change the UAV *U2* in this gene into a random UAV *U1* to generate *Pro-Chromosome P*. Since all UAVs encoding in *Pro-Chromosome P* are *U1*, then the second gene, which is selected randomly, in *Pro-Chromosome P* is changed to *U2*, which is different from the original number *U1*, thereby the mutated *chromosome Pʹ* is generated.

**Fig 12:**
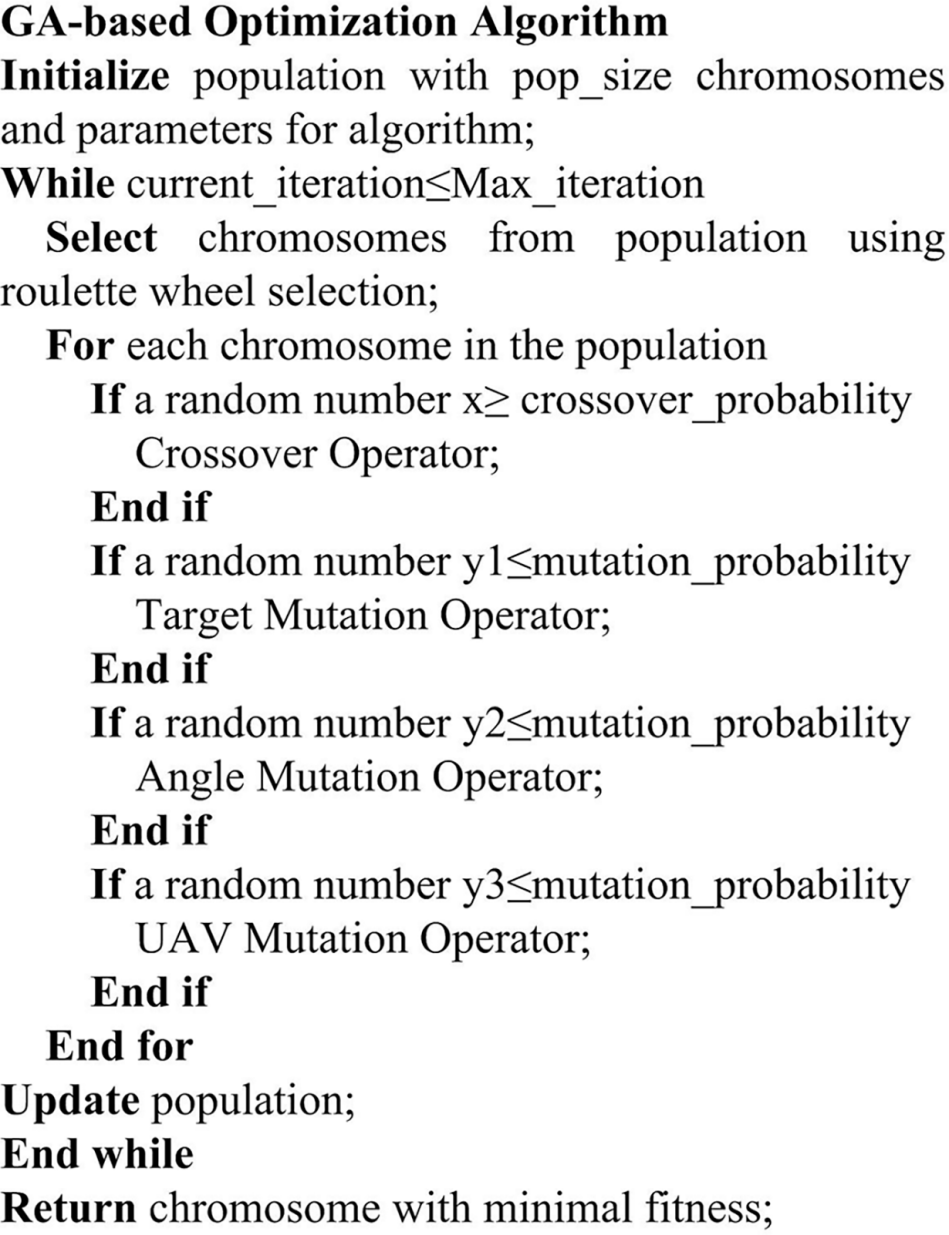
The pseudocode of the GA-based optimization algorithm.

The pseudocode of the GA-based optimization algorithm is shown in Fig 12.

## Illustrative example

**Fig 13:**
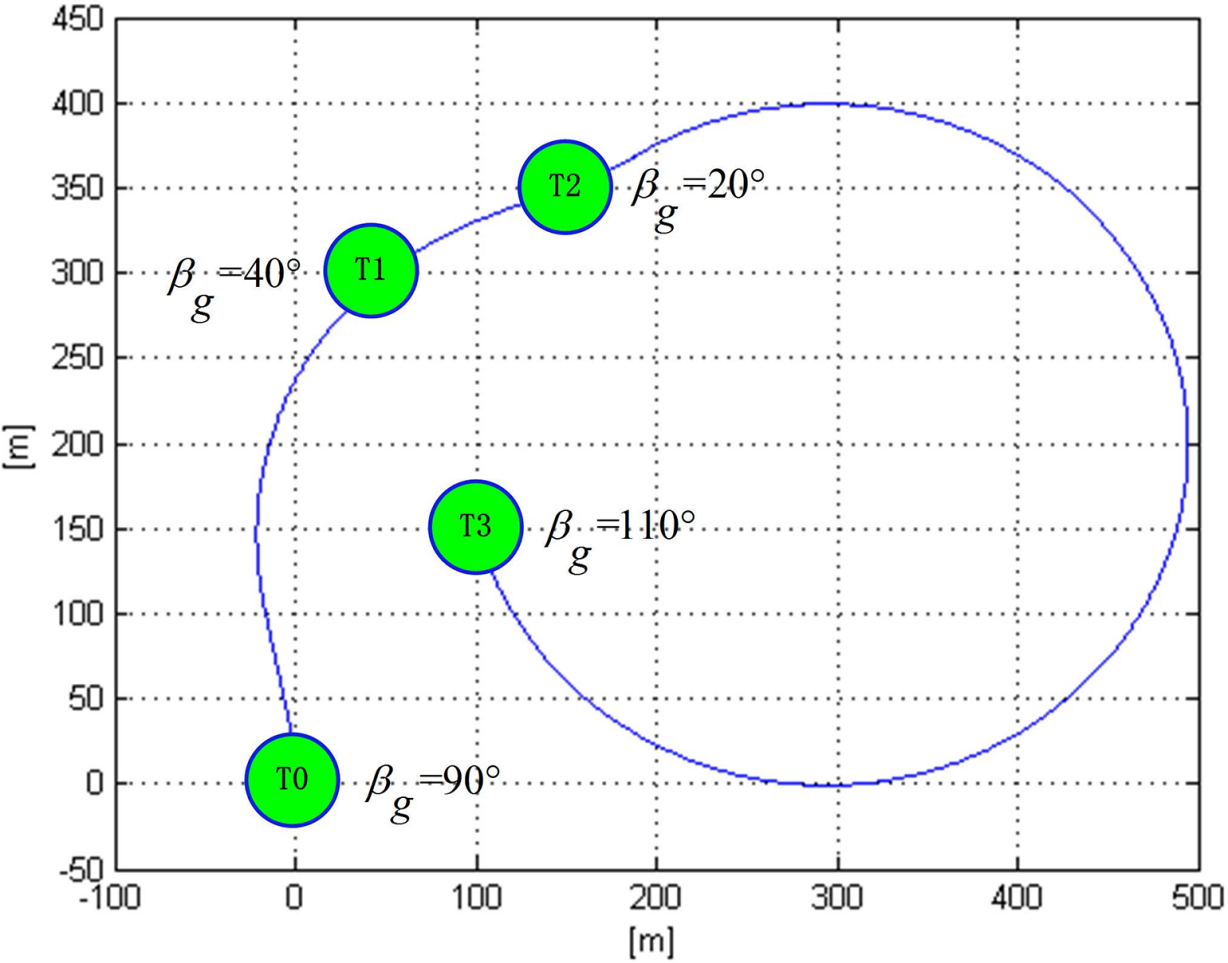
Optimal flight path for visiting three targets in a windless environment.

In the case of Ref. [9], the shortest path in which a UAV visited three targets, i.e., *T*_1_ (50, 300), *T*_2_ (150, 350), and *T*_3_ (100, 150), was 1483 m. The optimal task allocation and flight route of which are shown in Fig 13.

As the impact of wind was not taken into consideration, it could be regarded as a windless environment in which the ground speed of UAV was equivalent to its airspeed. When the airspeed was 10 m/s, the flight time of the UAV in the path of $\overset{}{\overset{⌒}{T_{0}T_{1}}}$, $\overset{}{\overset{⌒}{T_{1}T_{2}}}$, and $\overset{}{\overset{⌒}{T_{2}T_{3}}}$ can be calculated according to formula (12). However, the ground speed of the UAV may not be the same as the airspeed due to the impact of wind. Under four different steady winds of south wind, west wind, north wind, and east wind, with the same wind speed of 5 m/s, the flight time along the above path can be calculated according to formula (12), as shown in Table 2. Therefore, it can be concluded that the flight time of a UAV along the same planned route varies under different winds.

**Table 2:** UAV flight times along a fixed path under different winds.

Wind	Path $\overset{}{\overset{⌒}{T_{0}T_{1}}}$	Path $\overset{}{\overset{⌒}{T_{1}T_{2}}}$	Path $\overset{}{\overset{⌒}{T_{2}T_{3}}}$	Total time (s)
**Windless**	32.3190	11.2486	104.7440	148.3
**West wind**	51.0336	16.4476	105.8227	173.3
**South wind**	48.0447	13.9062	203.1994	265.2
**East wind**	28.3836	13.0658	140.5128	182.0
**North wind**	33.6465	12.6184	72.1318	118.4

Furthermore, there are six different orders for a UAV to reach the three above-mentioned targets, the optimal task allocation results and the shortest flight distance are provided in Ref. [9]. In the light of the above analysis, the flight time of UAV in six different paths under windless and four other different winds (east wind, south wind, west wind, and north wind, with a wind speed of 5 m/s) can also be calculated, as shown in Table 3. In the windless, west wind, east wind, and north wind, the shortest flight time for a UAV to reach the three targets are, respectively, 148.3, 173.3, 182.0, and 118.4 s, the task allocations scheme of which are all P2, while in the south wind environment, the shortest flight time for a UAV to reach the three targets is 165.6 s with a task allocation scheme P6. Obviously, task allocation of a UAV’s shortest flight time varies under different winds.

**Table 3:** UAV flight time in different visiting orders and different winds.

Scheme	Visiting order and ground speed heading angle	Minimum path length (m)	Flight time in Ref. [9] (windless) (s)	Flight time in west wind (s)	Flight time in south wind (s)	Flight time in east wind (s)	Flight time in north wind (s)
**P1**	T1(110°)→T2(50°)→T3(90°)	1667	166.7	356.6	251.7	308.6	293.9
**P2**	T1(40°)→T3(20°)→T2(110°)	1483	148.3	173.3	265.2	182.0	118.4
**P3**	T2 (250°)→T1(20°)→T3(30°)	2553	255.3	424.0	245.0	460.4	447.5
**P4**	T2(280°)→T3(200°)→T1(210°)	2512	251.2	349.9	233.4	546.8	503.5
**P5**	T3(40°)→T2 (110°)→T1(110°)	1583	158.3	294.7	260.0	395.6	268.2
**P6**	T3(40°)→T1(310°)→T2(180°)	1720	172.0	365.9	165.6	520.3	463.8

**Fig 14:**
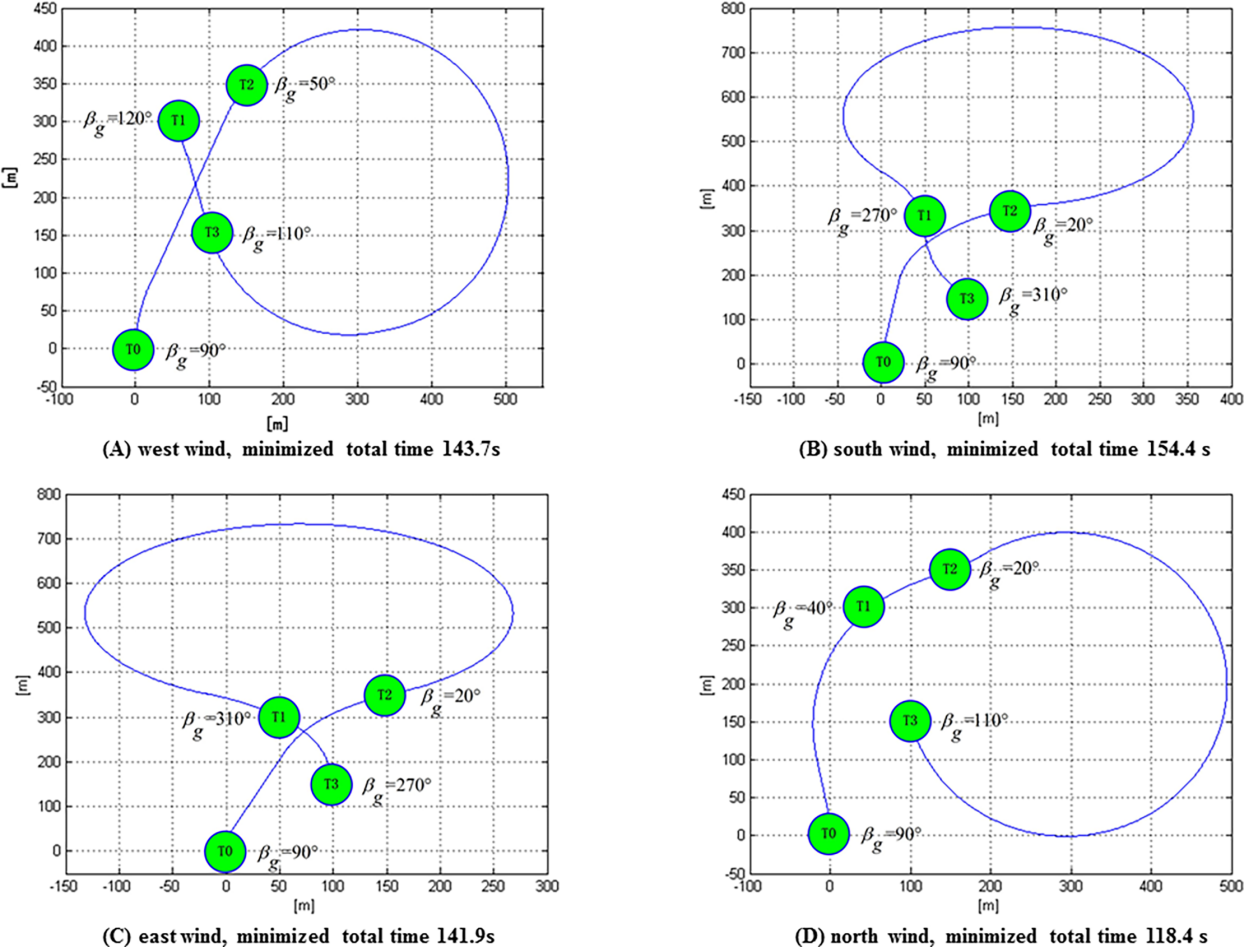
Flight path using minimum time for U1 to visit three targets under four different winds.

In fact, the task allocation schemes provided in Table 3 are the different choices for a UAV to reach the three above-mentioned targets with shortest flight time in the windless environment. However, the assigning of task and path planning of a UAV in the wind is not merely a choice among these six schemes in the windless environment, but rather a re-optimization in consideration of the impact of wind on the flight time. According to the VS-DP-VRP model built for this study, the shortest flight time and its corresponding optimal task allocation results of a UAV under four different winds (east, south, west, and north, with a wind speed of 5 m/s) can be calculated based on the GA-based optimization algorithm, as shown in Fig 14. For instance, in south wind with a wind speed of 5 m/s, the shortest flight time is 154.4 s and the optimal task allocation result is T3(20°), T2(270°), and T1(310°). Although this scheme is not included in Table 3, it is better than the overall schemes in Table 3.

## Experiments

For the integrated optimization of UAV task allocation and path planning under steady wind, simulation experiments of multi-UAVs visiting multi-targets are carried out, aiming at analyzing the shortest time for all of the UAVs to finish the task of visiting the targets and return to the starting point. All of the experiments are run on MATLAB on a 3.4-GHz CPU with 4 G memory; the detailed parameters of the experiments are shown in Table 4. Since the fixed-wing UAVs took off from the airport at the starting point, taxiing of UAVs would be restricted by the airport runway, as a result of which the ground speed heading angle in the experiment must be 90° when UAVs depart and return; otherwise, the UAVs cannot land on the runway.

**Table 4:** Parameter settings.

Parameter	Value
**Airspeed *V***_***g***_	10 m/s
**Minimum turning radius *r***_**min**_	200 m
**Discretization of ground speed heading angle, *N***_***g***_	36
**Starting point coordinates of S**	(0,0)
**Ground speed heading angle *β***_***g*0**_ **at starting point**	90°
**Targets with coordinates**	T1(50,300) T2(150,350) T3(100,150)

In order to determine the optimal allocation of the crossover probability and mutation probability in the algorithm, all of the possible parameter combinations are tested. Among them, the crossover probability ranges from 0.5 to 0.9, the mutation probability ranges from 0.1 to 0.5, and the population sizes for these ranges are 200, 400, 600, and 800. In this experiment, two UAVs are in the east wind with a wind speed of 5 m/s, and the generation of the algorithm was 200. Since there are some random uncertainties in the process of calculating the optimal solution, the result of each experiment might be different. Therefore, all the experimental results are the average of 20 experiments under the same experimental parameters.

**Fig 15:**
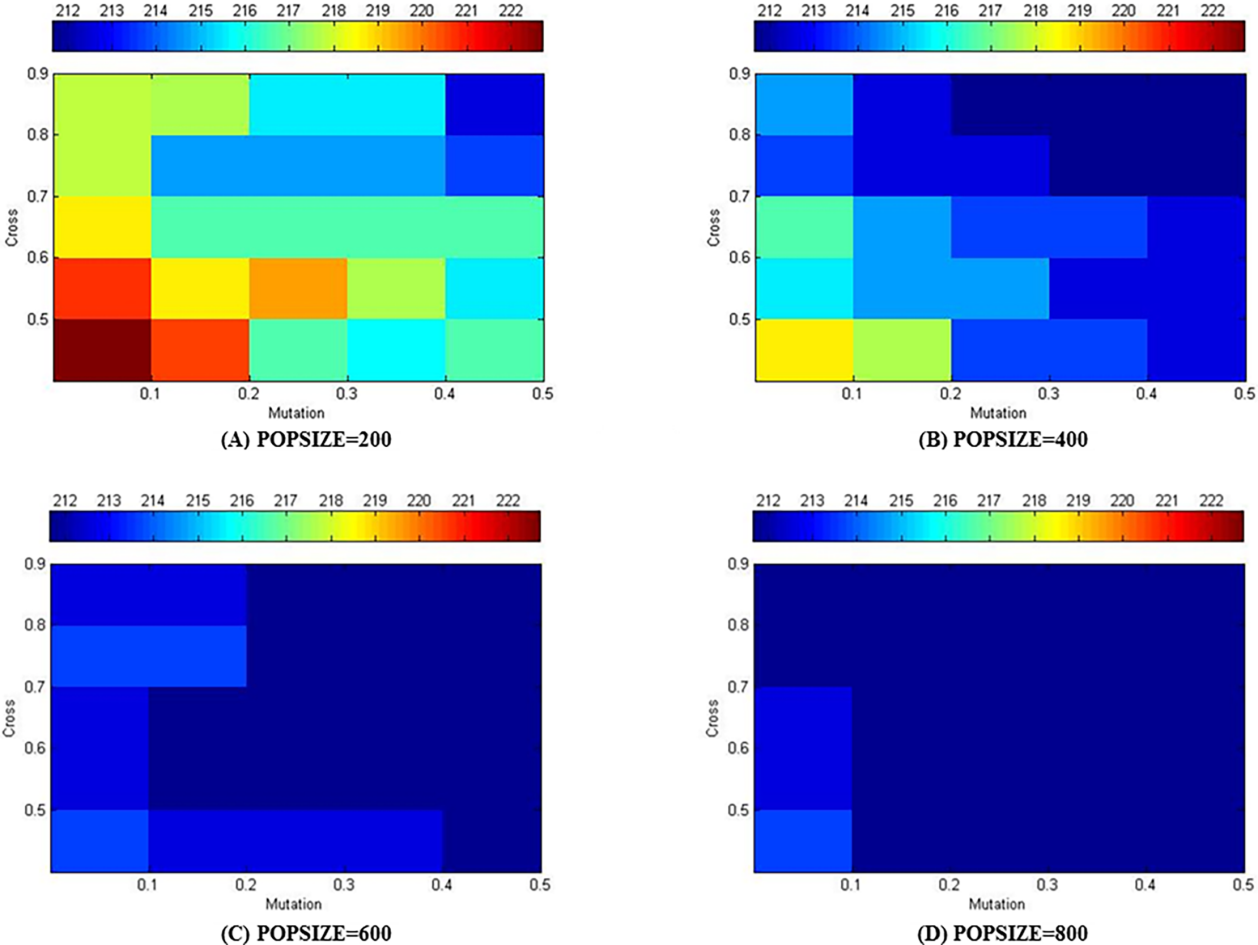
Minimum time in east wind field with wind speed of 5 m/s obtained by using different population sizes and crossover and mutation probabilities “Table in S1 Table”.

With different population size, crossover probability, and mutation probability, the result for the shortest time solved by the algorithm is shown in Fig 15. As the population size increased, the shortest time with the same crossover probability and mutation probability gradually decreased. Under a fixed population size, if the population size is small, the average of the shortest time after iterating the same number of times is different under different crossover and mutation probability, and if the population size is large, the average of the shortest time differed slightly after iterating the same number of times under different crossover and mutation parameter configurations and it is close to the optimal solution of the problem. As a result, the crossover and mutation probability has some relationship with the population size on the results on the algorithm.

**Fig 16:**
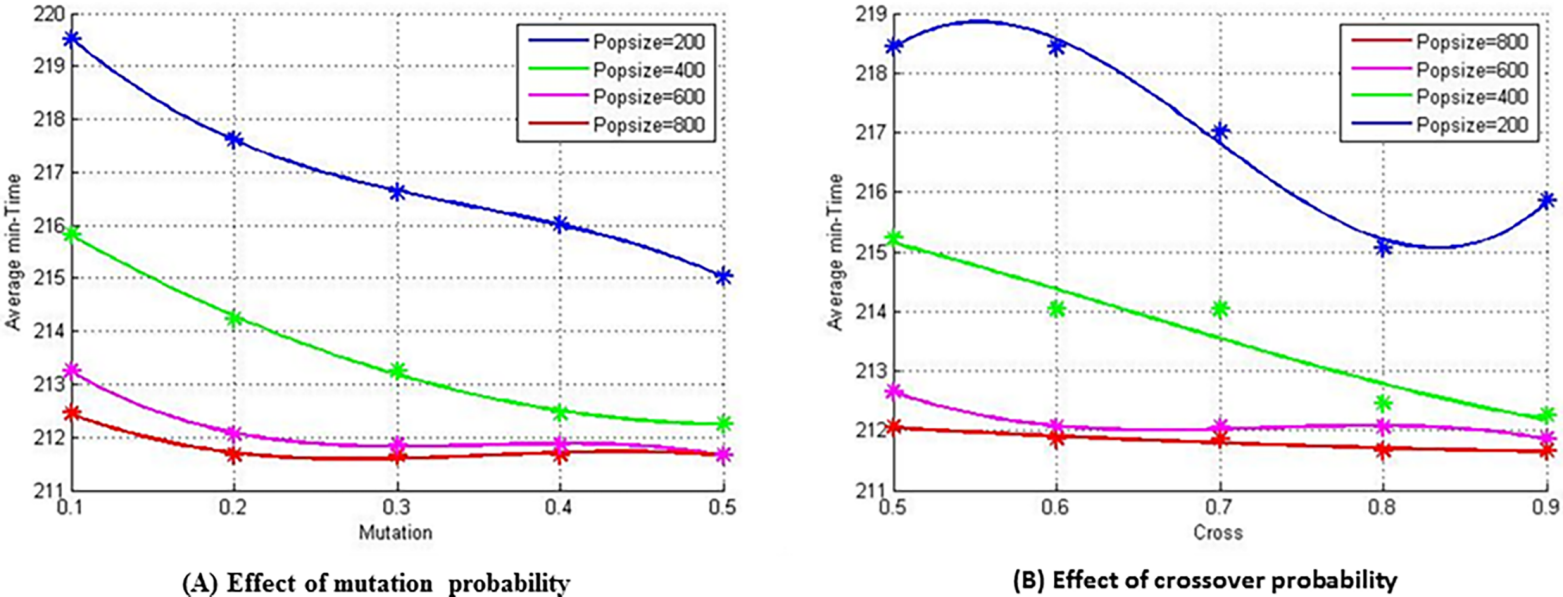
Effect of crossover and mutation probabilities on the results of the algorithm under different population sizes “Table in S2 Table”.

In the same scenario, 20 experiments are performed for each crossover probability, mutation probability and population size. When the mutation probability and the population size are fixed and the crossover probability is 0.5, 0.6, 0.7, 0.8, and 0.9, there are totally 100 experimental results, the average of which is defined as the average minimum time under the given configuration. Fig 16(A) shows the average minimum time with the variation of population size at different mutation probabilities. It can be concluded that the result becomes better with increasing mutation probability under different scales, which is more obvious when the population size is smaller. While the population reached a certain scale, the solutions became less sensitive to the mutation probability. With the same experimental method, the average minimum time with the change of population size at different crossover probability can also be obtained. The average minimum time in Fig 16(B) are also relatively sensitive to the population size, and better results can be obtained as the population size increases. However, the crossover probability has little effect on the solutions under larger populations.

Therefore, it can be concluded that the crossover and mutation probabilities have a positive relationship with the effect of the algorithm results; that is, the larger the value of the crossover or mutation probability, the smaller the value of average minimum time. When the population size exceeds a certain scale, the crossover and mutation probabilities still has a positive effect on the algorithm, but the influence is obviously reduced.

**Fig 17:**
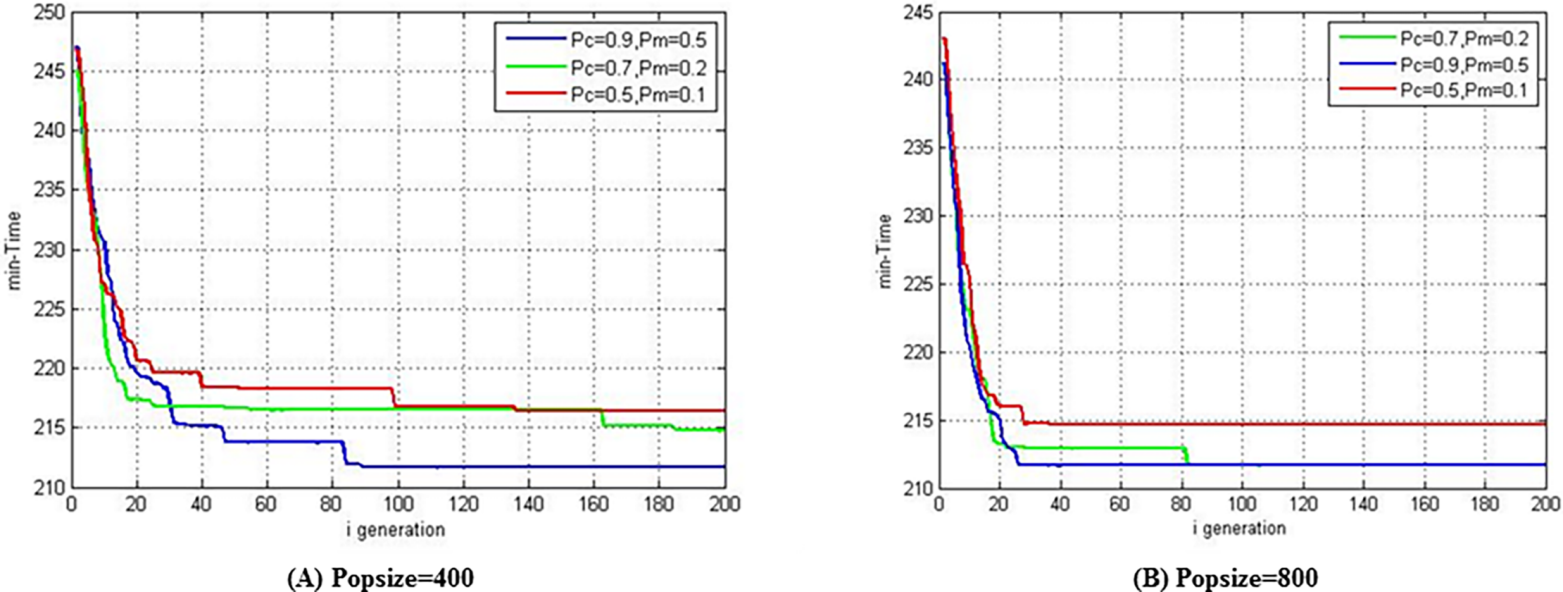
Algorithm solving process when given two population sizes and three kinds of crossover and mutation probability configurations “Table in S3 Table”.

In order to intuitively reflect the difference of the algorithm results in different populations and for different crossover and mutation probabilities, the three configurations of crossover and mutation probabilities are selected for population sizes of 400 and 800. 20 experiments are carried out under the same experimental configurations, and the results of the average minimum time, which varies with generation, is shown in Fig 17. When the population size is 400, the results of the three configurations are significantly different, and when the crossover probability is 0.9 and the mutation probability is 0.5, the algorithm has its best performance. When the population size increased to 800, the results among the three configurations are not significant difference, especially when the generation exceeds 90. It should also be noted that smaller population size can reduce the time cost of the algorithm’s operation at the same generation. After a comprehensive consideration of the above experimental process and results, in following experiments the crossover probability will be set to 0.9, the mutation probability to 0.5, the population size to 400, and the generation to 200.

On this basis, the method described in Ref. [9] and that outlined in this study are both adapted to analyze the minimum time during which two UAVs finished their task of visiting the targets and returning to the starting point under four different winds (south wind, west wind, north wind, and east wind, with a constant wind speed of 5 m/s), and the results are shown in Table 5.

**Table 5:** Results of task allocation and path planning under four different winds.

Wind (*V*_w_ = 5*m*/*s*)	Ref. [9]			VS-DP-VRP			Percentage of time saved by our method compared to Ref. [9] (%)
	Visiting order and ground speed heading angle	Flight time (s)	Minimum time to complete the task (s)	Visiting order and ground speed heading angle	Flight time (s)	Minimum time to complete the task (s)	
**West wind**	(U1,T2,90°) (U2,T3,130°)→ (U2,T1,90°)	228.3807 315.5454	315.5454	(U1,T3,220°) (U2,T1,40°)→ (U2,T2,20°)	222.0900 201.5115	222.0900	29.68
**South wind**	(U1,T2,90°) (U2,T3,130°)→ (U2,T1,90°)	287.4010 440.0221	440.0221	(U1,T1,80°)→ (U1,T3,50°) (U2,T2,320°)	229.8906 321.0472	321.0472	27.04
**East wind**	(U1,T3,210°) (U2,T1,100°)→ (U2,T2,60°)	318.0227 346.3122	346.3122	(U1,T3,330°) (U2,T1,40°)→ (U2,T2,20°)	211.6616 179.1433	211.6616	42.57
**North wind**	(U1,T1,90°) (U2,T3,70°)→ (U2,T2,70°)	233.2217 328.9634	328.9634	(U1,T3,30°) (U2,T1,40°)→ (U2,T2,20°)	178.6115 177.7637	178.6115	40.93

It can be concluded that, the actual flight time of UAV will be changed greatly along the path which is planned without consideration of the influence of wind. For the method proposed in this paper, the allocation of tasks and the planning of the route are integrated and optimized under the influence of the wind, the result of which is the best possible solution in the wind. Compared to the results in Ref. [9], the actual flight time will be reduced by 35.06% in the wind.

**Fig 18:**
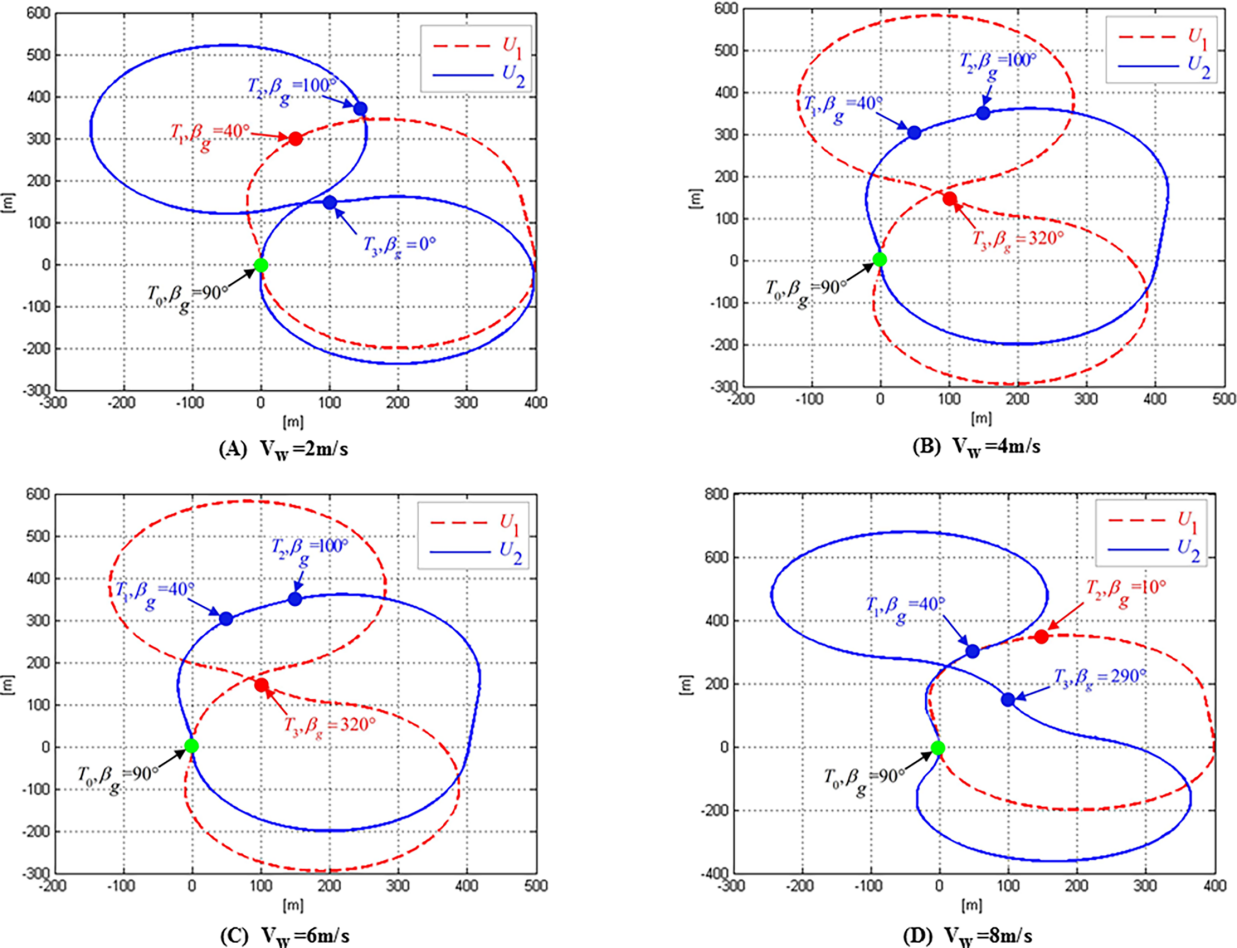
Minimum time paths for two UAVs to visit three targets in east wind with different wind speeds.

Furthermore, experiments are performed with different wind speeds ranges from 1 to 8 m/s in the east wind, the results of which are shown in Table 6. Similarly, the actual flight time in the wind will be reduced by 36.86%. The flight routes when the wind speed is 2, 4, 6 and 8 m/s are shown in Fig 18.

**Table 6:** Results of task allocation and path planning in East wind at different wind speeds.

East wind	Ref. [9]			VS-DP-VRP			Percentage of time saved by our method compared to Ref. [9] (%)
	Visiting order and ground speed heading angle	Flight time (s)	Minimum time to complete the task (s)	Visiting order and ground speed heading angle	Flight time (s)	Mnimum time to complete the task (s)	
***V***_**w**_ **= 1*m*/*s***	(U1,T3,210°) (U2,T1,100°)→ (U2,T2,60°)	269.0593 306.3960	306.3960	(U1,T3,0°) (U2,T1,40°)→ (U2,T2,20°)	240.5580 160.3431	240.5580	21.49
***V***_**w**_ **= 2*m*/*s***	(U1,T3,210°) (U2,T1,100°)→ (U2,T2,60°)	289.3673 308.9027	308.9027	(U1,T1,40°) (U2,T2,100°)→ (U2,T3,0°)	154.4964 236.5182	236.5182	23.43
***V***_**w**_ **= 3*m*/*s***	(U1,T3,210°) (U2,T1,100°)→ (U2,T2,60°)	312.0202 315.5552	315.5552	(U1,T3,340°) (U2,T1,40°)→ (U2,T2,20°)	221.6228 164.5546	221.6228	29.77
***V***_**w**_ **= 4*m*/*s***	(U1,T3,210°) (U2,T1,100°)→ (U2,T2,60°)	312.2888 327.3572	327.3572	(U1,T3,340°) (U2,T1,40°)→ (U2,T2,20°)	215.5220 170.1632	215.5220	34.16
***V***_**w**_ **= 5*m*/*s***	(U1,T3,210°) (U2,T1,100°)→ (U2,T2,60°)	318.0227 346.3123	346.3123	(U1,T3,330°) (U2,T1,40°)→ (U2,T2,20°)	211.6616 179.1433	211.6616	38.88
***V***_**w**_ **= 6*m*/*s***	(U1,T3,210°) (U2,T1,100°)→ (U2,T2,60°)	331.5437 376.5092	376.5092	(U1,T3,320°) (U2,T1,40°)→ (U2,T2,20°)	210.4490 193.2649	210.4490	44.11
***V***_**w**_ **= 7*m*/*s***	(U1,T3,210°) (U2,T1,100°)→ (U2,T2,60°)	358.3690 427.3303	427.3303	(U1,T3,310°) (U2,T1,40°)→ (U2,T2,20°)	212.2751 216.4469	216.4469	49.35
***V***_**w**_ **= 8*m*/*s***	(U1,T3,210°) (U2,T1,100°)→ (U2,T2,60°)	414.3254 517.9991	517.9991	(U1,T2,10°) (U2,T1,40°)→ (U2,T3,290°)	222.2152 239.7644	239.7644	53.71

## Conclusions and future works

Wind is a vitally important external factor in the actual flight of UAV. Different wind speeds and wind directions cause changes in heading angle and ground speed of UAV in actual flight. In this paper, in order to integrated optimize the task allocation and path planning of fixed-wing UAV, the steady wind environment was introduced into the optimization model, and the VS-DP-VRP model was established considering the dynamic constraints of UAV. The optimization objective of this model is to minimize the time required by UAVs to complete all of the tasks. Thus, the distance between any two targets was described by a Dubins path, and a method was proposed that combines the wind speed with the airspeed of UAV to calculate its ground speed. Considering that the VS-DP-VRP is still a NP-hard problem, the GA was chosen to solve this problem. The crossover operator in the GA and three kinds of mutation operators—namely, the mutation of targets, ground speed heading angle number mutation, and mutation in UAV allocation—were redesigned based on the characteristics of this problem. In addition, the feasibility and validity of the method were analyzed by an illustrative example, and the sensitivity of the influence caused by parameters such as population size, crossover probability, and mutation probability on the algorithm was analyzed. Moreover, the problem of multiple UAVs visiting multiple targets were comparatively analyzed, the results of which show that the proposed model and its algorithm can effectively provide a UAV task allocation and path planning scheme under steady wind. The wind direction and wind speed can be regarded as a steady wind as they are generally the same within a certain area. Yet changes in wind speed and wind direction cannot be ignored when the area is further expanded. In the future research, we will further consider the cases where wind speed and wind direction are constantly changing. Combined with UAV flight control strategy in variable wind, the integrated optimization of UAV task allocation and path planning under the influence of wind will be further explored.

## Supporting information

S1 Table Minimum time in east wind field with wind speed of 5 m/s obtained by using different population sizes and crossover and mutation probabilities. (XLSX)

S2 Table Effect of crossover and mutation probabilities on the results of the algorithm under different population sizes. (XLSX)

S3 Table Algorithm solving process when given two population sizes and three kinds of crossover and mutation probability configurations. (XLSX)
